# Bio-Based Smart Packaging Materials for Next-Generation Food Systems

**DOI:** 10.3390/ma19112393

**Published:** 2026-06-04

**Authors:** Ziao Zhang, Haowen Qian, Chun Shen, Shuping Wu

**Affiliations:** Institute of Polymer Materials, School of Materials Science & Engineering, Jiangsu University, Zhenjiang 212013, China; 3220708134@stmail.ujs.edu.cn (Z.Z.); 3230708053@stmail.ujs.edu.cn (H.Q.); 3230708002@stmail.ujs.edu.cn (C.S.)

**Keywords:** bio-based functional materials, smart packaging, cellulose and its derivatives, chitosan, sustainable food systems

## Abstract

Traditional petroleum-based packaging suffers from pollution and functional limits, making it unsuitable for next-generation food systems. In contrast, bio-based smart packaging—combining renewable substrates with responsive components—transforms packaging from a passive shell into an active quality monitor and supply chain information node through three interconnected pillars: renewability, real-time responsiveness to freshness markers, and digital traceability. Market figures confirm this shift, with the smart food packaging sector projected to reach USD 48.97 billion by 2028 (CAGR 4.49% from 2023). This review covers recent progress in natural polymers (cellulose, chitosan, alginate, gelatin) and bio-based polyesters (PLA, PHA). Their multiscale structures enable tunable mechanical and barrier properties while serving as hosts for intelligent functions. Two functional directions stand out: active preservation (antimicrobial, antioxidant, gas-regulating, stimulus-controlled release) and intelligent sensing (colorimetric indicators, bio-based sensors, nano-amplified signals for real-time freshness monitoring). Beyond material functions, digital tools such as IoT and blockchain turn packaging into interactive data nodes, linking material intelligence with full traceability to enhance food safety and supply chain efficiency. Key challenges remain with long-term operational stability, production costs, scalable manufacturing, and life cycle assessments. Nevertheless, bio-based smart packaging is expected to evolve through biomimetic design, process innovation, and system-level integration toward adaptability, multifunctionality, and intelligence, ultimately supporting safer, more transparent, efficient, and sustainable food systems.

## 1. Introduction

Packaging has evolved from a traditional food protection carrier to a core functional node within the new generation of food systems, with its performance directly impacting the overall efficiency of the system [1,2,3,4,5,6]. The global food system faces interconnected structural challenges that compromise its sustainability and safety. Significant food loss and waste persist, alongside ongoing safety risks such as microbial contamination and the improper use of additives, which complicate effective prevention and control measures [7,8,9,10,11]. Furthermore, the widespread use of conventional fossil-based packaging contributes substantially to carbon emissions, intensifying environmental pressures. These issues are compounded by pervasive information asymmetry across supply chain stages, leading to a systemic lack of transparency that impedes traceability and undermines accountability [12,13].

The traditional fossil-based packaging is limited not only by resource depletion and environmental pollution but also by functional constraints—their passive nature, only providing basic physical barriers and limited spoilage protection, fails to meet the dynamic monitoring and regulatory requirements of modern food system [14,15,16,17,18]. Even conventional bio-based packaging often also lacks integrated intelligent responsiveness, precluding real-time quality traceability and early risk warning. Accordingly, next-generation smart packaging systems, which feature sustainability, intelligence, resilience, and digital integration, have emerged as a critical research frontier, emphasizing resource circularity, proactive risk prevention, anti-interference capability, and seamless information interoperability [19,20,21,22].

Bio-based smart packaging integrates proactive data generation with inherent sustainability, combining renewable, non-toxic, and biodegradable bio-substrates with the sensing, signaling, and data functions of smart components, thus serving as key proactive functional elements in future food systems. Smart packaging enables non-contact monitoring of food and environmental quality to reduce spoilage losses, while active packaging focuses on substance release or scavenging to extend shelf life—distinct concepts often integrated in practice [7,23,24,25,26,27]. By detecting spoilage gases, temperature, and humidity, these systems generate visual or quantifiable data to optimize supply chains, enhance quality control, and support consumer decision-making. Specifically, they incorporate active components (e.g., antimicrobials, antioxidants) for active preservation and use pH-, gas-, or temperature-responsive mechanisms for in situ freshness indication. When further integrated with digital tools, such as IoT and blockchain, the packaging acts as a data node within the supply chain, offering an integrated solution to curb waste, enhance safety, and improve traceability [28,29]. The market potential for these systems is substantial, with the smart food packaging sector projected to reach a value of USD 48.97 billion by 2028, reflecting a compound annual growth rate (CAGR) of 4.49% from 2023 to 2028 [7]. A diverse range of bio-based materials supports this growth, including wood-derived polymers (e.g., cellulose, lignin), protein-based polymers (e.g., gelatin, whey protein, soy protein), and microorganism-derived polymers (e.g., polyhydroxyalkanoates (PHAs), polylactic acid (PLA)), which are intensively explored as sustainable alternatives to conventional packaging [30,31].

**Figure 1:**
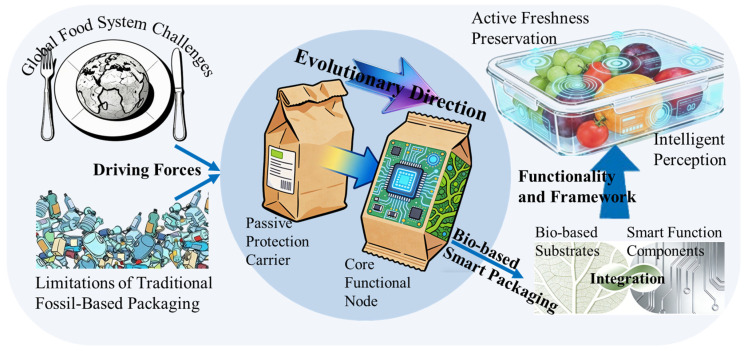
The passive protection provided by traditional fossil-based packaging is contrasted with the core functional nodes of bio-based intelligent packaging, driving the evolution direction towards the next-generation food system: bio-based substrates, active preservation, intelligent components, and intelligent sensing. (The decorative elements and icons in Figure 1 depicting intelligent packaging and active freshness preservation concepts were generated using AI tool (Doubao, Doubao-Seed 2.0)).

As a sustainable alternative to fossil-based materials in food systems, bio-based smart packaging has garnered increasing research and application attention. Previous studies have confirmed the effectiveness of various bio-based and intelligent strategies in enhancing food safety and reducing environmental impact, while also highlighting the promising role of nanomaterials in this field [32,33,34,35,36,37]. Nevertheless, critical scientific and technical questions remain unresolved. These span from elucidating the structure–property relationships of foundational materials (e.g., cellulose, chitosan, PLA) and achieving the synergistic, stable performance of active functions, to the precise integration and real-world adaptation of smart sensing technologies—all of which demand further clarification to overcome current technical bottlenecks. Against this backdrop, this review focuses on bio-based smart packaging materials for next-generation food systems. This paper first reviews the composition and classification of key materials, analyzes the structure–property relationships within their multiscale material platforms, and evaluates the characteristics, advantages, and limitations of these functional materials. Building upon this foundation, it systematically elucidates the core functional mechanisms enabling active preservation and intelligent response in these materials. Subsequently, it explores pathways for integrating smart sensing technologies with digital systems, examines the enabling role of nanotechnology, and reviews application potential across diverse scenarios, such as fresh produce and convenience foods. Finally, it dissects current technical, economic, and regulatory challenges while projecting future trends toward biomimetic, adaptive, and system-integrated developments. This comprehensive analysis aims to provide systematic guidance for academic innovation and industrial applications in this field. Figure 1 depicts the positioning of bio-based smart packaging in next-generation food systems, illustrating an evolutionary pathway that begins with addressing traditional packaging limitations, advances through integrated innovation in materials and functionality, and ultimately achieves a transformation into a key enabling node within the food system.

## 2. Bio-Based Material Platforms for Smart Packaging

### 2.1. Natural Polymers and Biopolymers

Natural and biopolymers, derived from renewable sources, form a fundamental material platform for smart packaging due to their inherent biocompatibility and versatile structural adaptability. Table 1 summarizes selected biopolymers that enable functionality through smart packaging.

#### 2.1.1. Cellulose and Its Derivatives

Cellulose and its derivatives represent a research hotspot in the field of food packaging, with studies focusing on optimizing functional derivatives and other modification techniques for waterproofing and oil resistance, thereby enhancing mechanical, barrier, and antimicrobial properties. As a linear polymer composed of D-glucose units, native cellulose possesses a dense hydrogen-bond network that provides high mechanical strength, thermal stability, and favorable barrier properties [38,39]. It should be kept in mind, however, that chemically modified cellulose derivatives—for instance, those produced via etherification, esterification, or graft copolymerization—can adopt branched or even hyperbranched architectures. Films derived from cellulose (typically from native or modified forms) exhibit excellent toughness, tensile strength, transparency, and optical performance. However, strong hydrophilicity limits their use in high-temperature or high-humidity environments [40,41,42,43,44,45]. To overcome this, covalent functionalization, through modifications like acetylation or salinization, is commonly employed to enhance water resistance and barrier properties, reduce leaching of components in humid conditions, and enhance compatibility with smart components [46,47].

Plasticizers such as glycerol form amorphous domains with cellulose through intermolecular hydrogen bonding, improving flexibility while retaining high light transmittance, high water vapor permeability, and low oxygen transmission rates—ideal for packaging high-respiration fresh produce [48,49,50,51]. Reinforcing fillers (e.g., lignin) boost mechanical strength and UV stability, while active additives (e.g., antioxidants, antimicrobials) extend shelf life and improve biosafety. Molecular design also enables hydrophobic and oil-resistant modifications of cellulose, balancing performance with biodegradability. Alternatively, multi-component composites can create multifunctional high-barrier materials with enhanced barrier properties. Inspired by nacreous architectures, a bio-based dual-layer composite film incorporating a natural indicator, polysaccharide, with a functional inner layer synergistically combines antioxidant and pH-responsive color-changing properties, enabling real-time visual freshness monitoring across various meats and showing strong potential for universal application [52,53].

Nano-cellulose derivatives, including cellulose nanofibers (CNFs) and cellulose nanocrystals (CNCs), offer high specific surface areas after micro/nano-processing, combining high tensile strength, excellent gas barrier properties, low permeability coefficients, and low thermal expansion coefficients, which make them suitable as reinforcing materials for sensing platforms in intelligent packaging [54,55,56,57]. Derivatives such as carboxymethyl cellulose (CMC) and hydroxypropyl methylcellulose (HPMC) undergo etherification modification to optimize water solubility and film-forming properties, yielding highly flexible, low gas permeability transparent films [58,59,60]. Blending with biopolymers further enhances smart functionalities [40,61]. Cellulose acetate (CA), enhanced through esterification for solvent resistance and hydrolysis stability, finds widespread use in rigid packaging [62,63]. Bacterial cellulose (BC), characterized by high purity, an ultrafine three-dimensional network structure, and high specific surface area, exhibits a highly developed pore and tunnel network conducive to diffusion of embedded active or smart materials, demonstrating excellent adaptability in specific packaging applications [64,65]. Ethyl cellulose (EC) and methyl cellulose (MC) also serve as key components in smart active packaging [66,67,68,69].

#### 2.1.2. Chitosan and Chitin Derivatives

Chitosan (CS), a deacetylated derivative of chitin, possesses abundant amino and hydroxyl groups that grant it inherent antibacterial activity, cationic polyelectrolyte behavior, and chemical versatility [70,71,72,73]. The antimicrobial effect mainly comes from protonation of the amino groups, which disrupts microbial cell membranes and suppresses common foodborne pathogens. Pure CS-based packaging suffers from high hydrophilicity, poor mechanical strength, and limited functionality. To overcome these drawbacks, chemical modification (e.g., quaternization, carboxymethylation) or composite strategies are widely adopted. Such approaches improve water solubility, film-forming capacity, and antioxidant/antimicrobial efficacy, while also enabling advanced functions like pH-responsive sensing and ion detection [74,75,76].

As a mainstream derivative of chitosan, carboxymethyl chitosan (CMCS) possesses excellent biocompatibility and film-forming capabilities, making it an ideal substrate for food packaging films [77,78,79,80]. Blending with polymers such as polyvinyl alcohol (PVA) or compositing with sodium alginate (SA) to form multilayer structures via layer-by-layer self-assembly improves flexibility, chemical resistance, barrier properties, and mechanical stability [81]. Such composites also facilitate the efficient loading and controlled release of antimicrobial and antioxidant agents, thereby inhibiting microbial growth and extending product shelf life. SA, as a natural anionic polysaccharide, interacts with CMCS through hydrogen bonding and electrostatic forces, further enhancing film performance [74]. For instance, multifunctional smart packaging films, fabricated by complexing and blending SA-proanthocyanidin (SA-PC) with CMCS-curcumin (CMCS-Cur), integrate pH-responsive colorimetric sensing, UV blocking, antibacterial, and antioxidant functionalities, along with favorable biodegradability, enabling simultaneous real-time freshness monitoring of shrimp and extended preservation of grapes [74]. Chitosan nanofibers (CSNFs) or nanoparticles (CSNPs) can also be incorporated as functional fillers into other polymer matrices to impart sustained antibacterial and antioxidant activity, delaying lipid oxidation and microbial contamination [82,83].

For functional expansion, CS-based active and smart films can be designed to respond to biomarkers such as ammonia and pH via embedded natural indicators (e.g., Anthocyanins (ACNs), Cur), enabling real-time visual monitoring of food freshness. Incorporating active compounds—such as phenolics for enhanced mechanical and barrier properties, or essential oils and plant extracts for strengthened antimicrobial and antioxidant functions—further tailors the film’s performance [84]. Essential oils disrupt microbial membranes and inhibit efflux pumps; encapsulation in biopolymer matrices allows controlled release and extends shelf life. Polyphenol-rich plant extracts scavenge free radicals and chelate metal ions, reinforcing the antimicrobial effect. Antimicrobial peptides can be immobilized onto CS films via electrostatic adsorption, enabling targeted antibacterial activity while retaining biocompatibility. However, the inherent hydrophilicity of chitosan leads to swelling in acidic or humid conditions, undermining mechanical strength and moisture barrier properties. Surface nanoengineering offers a potential route to improve hydrophobicity by increasing the water contact angle [85]. Current challenges for CS-based smart packaging include limited sensitivity to early spoilage indicators and a narrow application scope, requiring further research for commercial adoption.

#### 2.1.3. Starch and Alginate

Starch and sodium alginate (SA) are abundant, low-cost, biodegradable, and non-toxic, as well as easy to process and form, making them suitable for direct food contact applications, and have garnered significant attention in the food packaging sector [86,87,88,89,90,91]. Starch possesses excellent gelatinization to form films, where amylose enhances packaging strength and crystallinity, while amylopectin improves flexibility [92,93,94,95,96]. Starches from different sources can be used alone or blended with other natural polymers to create biodegradable coatings or packaging films that extend food shelf life [97]. For instance, corn starch/gelatin–sorbitol composite coatings improve post-harvest quality in grapes, while mango kernel starch packaging extends tomato shelf life. However, starch-based packaging exhibits strong hydrophilicity, often resulting in poor barrier and mechanical properties, high brittleness, and insufficient moisture resistance. Modification with plasticizers is frequently required to reduce deformation stress [98,99]. Alternatively, functional natural extracts (such as beetroot extract) are incorporated as bio-based sensors, not only reducing light transmittance and water vapor permeability but also enhancing flexibility and hydrophobicity via intermolecular interactions; concurrently, it confers pH responsiveness for freshness monitoring [99,100].

SA can form gels through ionic crosslinking, making it suitable for microencapsulating active compounds and constructing smart devices that indicate and track food freshness. Pure SA films exhibit low mechanical strength, susceptibility to cracking, humidity sensitivity, poor barrier properties, and limited protection against flavor compounds, which can be improved through nanocomposites, chemical crosslinking, blending with other polysaccharides (e.g., CS, pectin, starch), and incorporating lipids or proteins [101,102,103,104,105,106,107].

#### 2.1.4. Protein-Based Materials

**Figure 2:**
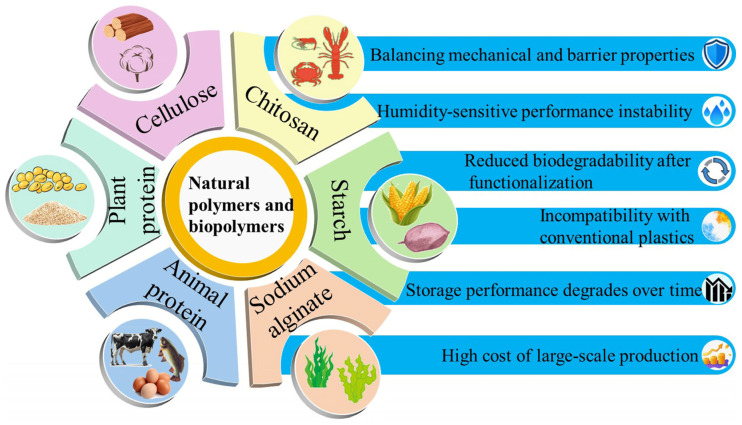
Classification and common challenges of natural polymers and biopolymers (cellulose from cotton/wood, chitosan from shrimp/crab, starch from corn/yam, animal protein from milk/fish/eggs, plant protein from soy/grains, sodium alginate from seaweed) in smart food packaging: mechanical/barrier trade-off, hygroscopicity, reduced degradability, plastic incompatibility, storage decay, and high cost. All biopolymers shown are derived from natural sources.

Plant-based (e.g., soy protein) and animal-based (e.g., gelatin) proteins form three-dimensional network structures through amino acid residues, exhibiting excellent film-forming properties, UV barrier performance, flexibility, transparency, and gas barrier properties [108,109,110]. Their high biocompatibility makes them suitable for direct-to-eat integrated packaging applications [111,112,113,114,115,116,117,118]. Their mechanical properties fall between hydrophilic polysaccharides and hydrophobic polyesters, balancing mechanical strength, barrier efficiency, and bioactivity [119]. For instance, gelatin composites inhibit foodborne pathogens [120,121,122], while soy protein films form cohesive networks through thermal or chemical denaturation, exhibiting superior oxygen barrier properties to polysaccharide and lipid films under dry conditions [123], and whey protein isolate coatings significantly delay lipid oxidation and hydrogen peroxide generation [124]. When combined with CS, they further enhance network structure, increase mechanical strength, and reduce water vapor transmission [125]. However, protein materials are susceptible to microbial degradation and exhibit poor acid/alkali resistance and moisture tolerance. Their preservation and antimicrobial functions require further enhancement through crosslinking, nanocomposites, or the addition of natural antimicrobial agents or phenolic extracts [126]. This ensures stability and maintains the freshness of perishable products for advanced packaging needs [119]. Figure 2 presents a simplified classification of natural polymers and biopolymers used in smart food packaging, along with a summary of their prevalent challenges.

**Table 1:** Representative biopolymers for functional smart packaging.

Materials	Active Ingredients	Applications	Products	Refs.
MC	Jambolao’s anthocyanins	Extend shelf life; indicate freshness.	Meat or seafood	[127]
MC-CSNF	Saffron anthocyanins	Monitor pH levels and volatile gas; real-time freshness monitoring.	Meat or seafood	[128]
CMC/starch	Purple sweet potato’s anthocyanins	Real-time monitoring of freshness; pH or NH_3_ level indicator.	Fish	[129]
BC	Garlic extract; bromophenol blue	Delay spoilage; pH indicator; monitor freshness.	Meat (e.g., beef)	[130]
BC	Pelargonidin	Visual monitoring of freshness.	Tilapia filets	[131]
CA	Cur	Thermochromism; temperature sensor to monitor food quality.	Entire supply chain	[132]
Guar gum, SA, and carboxylated cellulose nanofibers	Black rice extract, curcumin, and their compounds	Bionic shellfish sensor; visual freshness monitoring.	Chicken, pork, and shrimp	[52]
Oxidized chitin nanocrystals/CS	Black rice bran anthocyanins	Monitoring the spoilage process; pH indicator.	Fish and shrimp	[133]
CMCS/SA	PC and Cur	Monitor freshness of shrimp; extend shelf life of grapes.	Shrimp and grapes	[74]
CS/PVA	Alizarin, 9-Phenanthreneboronic acid, and beeswax	Anti-counterfeiting; food freshness preservation and visual monitoring of spoilage; extend shelf life.	Fish	[85]
CS/pectin	Nystatin and epigallocatechin gallate	Slow microbial growth and quality deterioration.	Strawberries	[134]
Starch/gelatin	Carrot anthocyanin	pH colorimetric indicators reveal meat spoilage.	Meat	[135]
Joha rice starch	Beetroot extract	Monitor food freshness.	Chicken, fish, and Indian cottage cheese	[99]
Pectin/SA/xanthan gum	Raspberry pomace extract	Monitor freshness of high-protein foods.	Pork rind and lard	[136]
SA-CS	Red prickly pear’s betalain	Monitor freshness.	Salmon	[137]
SA-Guar gum	Glucose–glycerol carbon dots	Monitor freshness through humidity changes (fluorescence intensity).	Bread	[138]
Fish gelatin/PVA	Vinasse anthocyanins	Freshness monitoring; packaging refrigerated foods	Shrimps	[139]
Gelatin/K-carrageenan	Anthocyanins and TiO_2_ nanoparticles	pH indication; antimicrobial and antioxidant; monitor freshness.	Fish	[140]
Soy protein isolate	CNC/Cur/polyvinylpyrrolidone nano-capsules	Monitor freshness.	Shrimp	[141]
Collagen	Laponite @Cur-CA	Monitor freshness; extend shelf life.	Shrimp	[142]

### 2.2. Bio-Based Polyesters and Emerging Platforms

Bio-based polyesters address the performance shortcomings of natural polymers for smart packaging through controlled molecular structures, which are achievable via microbial fermentation or chemical synthesis and have exceptional stability, positioning them as crucial enabling materials.

#### 2.2.1. Polylactic Acid and Polyhydroxyalkanoates

Polylactic acid (PLA) and polyhydroxyalkanoates (PHAs) represent the principal bio-based polyesters in food packaging [143,144,145,146,147,148,149,150,151]. PLA, synthesized from renewable resources such as corn starch or sugarcane via fermentation and ring-opening polymerization, exhibits high transparency, mechanical strength, compostability, biocompatibility, and full biodegradability within months under industrial composting conditions, which make it an ideal sustainable alternative to traditional plastics [152,153]. Its tensile strength approaches that of polyethylene (PE) (suitable for rigid packaging), and its oxidation resistance rivals most conventional plastics without the risk of harmful substance migration [154,155]. However, its applications are constrained by inherent brittleness, low thermal resistance, high permeability, and relatively high production costs, with its low melting point further restricts its application in high-temperature food packaging [156,157]. Consequently, modification strategies for PLA include additive incorporation, polymer blending, nanocomposite formation, surface coating, and crystallinity control [153,158]. For instance, incorporating modified starch enhances tensile strength and overall barrier performance, surpassing both pure PLA and conventional petroleum-based materials. Nanocomposites and surface treatments further improve structural integrity, oxygen barrier properties, moisture resistance, and antimicrobial functionality [159,160]. Annealing processes can optimize crystallinity, thereby improving heat resistance and barrier performance, making PLA suitable for hot-food packaging [161]. Currently, recycling approaches are evolving from traditional degradation toward high-value conversion pathways like catalytic, photocatalytic, and bio-enzymatic pathways to enable sustainability in packaging [162,163].

PHAs, encompassing polyhydroxybutyrate (PHB), polyhydroxy-valerate (PHV), and others, are polyesters produced by microbial fermentation of sugars, lipids, or oils [153]. They demonstrate excellent biocompatibility, moisture resistance, gas barrier properties, tunable mechanical, thermal stability, and thermoplasticity. Their performance can be further optimized through adjusting monomer compositions. Unlike PLA, which requires industrial composting for degradation, PHAs degrade fully in diverse natural environments—such as soil, oceans, and wastewater—making them particularly suitable for marine-biodegradable eco-friendly packaging [153,164,165]. They also generally have higher melting points than PLA, making them more suitable for hot-food packaging and high-heat barrier applications [166]. High production costs remain a challenge, yet mixed microbial culture (MMC) fermentation offers a potential path to cost reduction [167].

Both PLA and PHAs function as core materials in smart packaging systems. By regulating crystallinity through blending or copolymerization, their mechanical and barrier properties can be tailored to integrate intelligent functions such as temperature responsiveness and gas sensing. Their complementary characteristics enable the design of multilayer packing films, where enhances barrier performance and mitigates PLA’s moisture sensitivity, broadening food packaging applications [168]. In the near term, PLA remains a cost-effective and readily available alternative, while PHAs, with their superior biodegradability and multifunctionality, are positioned for long-term sustainable use—especially under demanding degradation conditions or higher operating temperatures [153].

#### 2.2.2. Emerging Bio-Based Polyesters

Emerging bio-based polyesters include poly (ethylene 2,5-furandicarboxylate) (PEF), synthesized from biomass-derived furan-dicarboxylic acid and ethylene glycol [169,170,171]. Structurally similar to conventional petroleum-based polyethylene terephthalate (PET), PEF uses renewable feedstocks, making it a promising bio-based alternative [172,173]. Compared to conventional bio-based polyesters, PEF exhibits significantly superior barrier properties, with O_2_ and CO_2_ permeability values 2–11 and 10–19 times lower than PET, respectively, and water permeability reduced by approximately half [174]. This advantage comes from the higher rigidity of the furan ring relative to terephthalate units, which restricts segmental motion and reduces molecular diffusion in the amorphous phase.

Although sharing comparable transparency, barrier performance, and mechanical strength with PET, PEF’s application is hindered by a slower crystallization rate and inherent brittleness in films and bottle packaging [175]. Low-load polymer nanocomposite modification leverages the high specific surface area of nanoparticles to simultaneously enhance PEF’s mechanical and crystallization properties while imparting innovative functions, such as UV shielding, blue light blocking, and antimicrobial properties [176,177,178]. Miah et al. [169] demonstrated that incorporating just 1.0wt% carboxylated cellulose nanofibers into the PEF matrix via hydrogen bonding can simultaneously enhance mechanical and barrier properties, enabling effective preservation under high temperature and high humidity. Furthermore, the chemical reactivity of the furan ring provides a platform for developing advanced, responsive smart packaging materials for food quality monitoring. Table 2 demonstrates performance enhancements in bio-based polyester smart packaging, achieved through specific material and functional modifications.

#### 2.2.3. Structure–Property Relationships Relevant to Smart Packaging Applications

**Figure 3:**
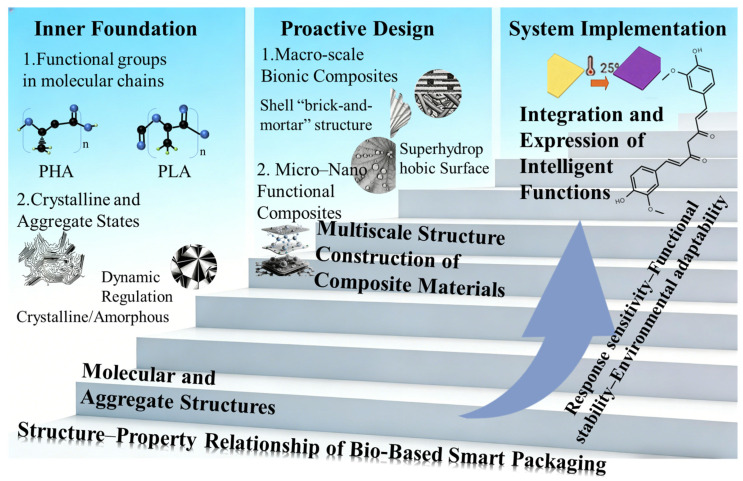
Schematic of the multiscale structure–property relationship in bio-based smart packaging, from molecular chain functional groups and aggregate states (crystalline/amorphous), through macroscopic biomimetic design and micro–nano functional composites, to system-level integration of intelligent functions. (The decorative elements and icons in Figure 3 depicting hierarchical progressive background were generated using AI tool (Doubao)).

The bio-based smart packaging performance is governed by a multiscale structural hierarchy that determines responsiveness, stability, and environmental adaptability. At the molecular level, functional groups (e.g., hydroxyls in PLA and PHAs) provide anchoring sites for smart components, directly influencing functional efficiency. Crystallinity serves as a key regulator of mechanical and barrier properties; however, while high crystallinity improves stiffness and barrier performance, it often compromises flexibility, necessitating precise modulation through blending, copolymerization, or annealing. The amorphous regions of PLA host more functional molecules, whereas the crystalline domains of PHA enhance response stability. Furthermore, interfacial interactions in composite systems critically govern structure–property synergies. For example, the addition of Cu promotes ordered crystalline arrangements, enhancing barrier performance and demonstrating the ability of functional additives to modulate polymer aggregation [184]. The multiscale structure–property relationships underlying bio-based smart packaging materials are depicted in Figure 3.

To further optimize performance, multiscale structural design is applied across different length scales. Macroscopically, biomimetic multilayer composite structures—inspired by the “brick-and-mortar” architecture of seashells—enhance barrier and mechanical properties by creating tortuous diffusion pathways through the oriented dispersion of rigid mica flakes in a PLA matrix [52]. A hydrophobic outer layer simultaneously shields a hydrophilic smart inner sensing zone, thereby overcoming the poor water resistance of conventional polysaccharide films. Similarly, the reed-leaf-inspired superhydrophobic surface increase water contact angles, achieving outstanding water repellency and anti-fouling properties [28]. At the micro–nano scale, integrating functional nanofillers (e.g., Co-based MOFs for ammonia detection, surface-modified Laponite (LAP) nanosheets for Cur delivery) via robust interfacial bonding ensures stable immobilization of active molecules, enabling high sensitivity, stability, and multifunctional performance [142]. The photophysical properties of functional molecules, like umbelliferone (UMB) and Cur, including pH-responsive and fluorescent mechanisms, are determined by their chemical structures, making their stable nano-integration essential for reliable smart-packaging performance.

## 3. Active Functions in Bio-Based Smart Packaging

### 3.1. Antimicrobial and Antifungal Functions

The antimicrobial and antifungal functionalities of bio-based smart packaging are essential for ensuring food safety and extending shelf life, achieved through either the intrinsic activity of endogenous antibacterial biopolymers or the integration of exogenous functional components [185,186,187,188,189,190]. This offers dual advantages of environmental sustainability and highly effective antimicrobial activity. Endogenous antibacterial biopolymers exhibit natural antimicrobial activity through their chemical structure, eliminating the need for additional antimicrobial agents, while offering high biocompatibility and avoiding secondary pollution. Typical examples, such as CS (protonated amino groups under acidic conditions disrupting the cell membrane integrity of common foodborne pathogens), BC (ultrafine 3D network acting as a physical barrier against microbial invasion while creating a microenvironment that inhibits microbial growth), and quaternized cellulose (inducing cell death through electrostatic interactions of cationic groups), provide biocompatible, non-leaching antimicrobial properties [191,192].

The synergistic integration of natural antimicrobial agents with functional nanomaterials within bio-based matrices enables significantly enhanced antibacterial efficiency, persistence, and stability. Natural antimicrobial agents, such as plant essential oils (rich in phenolic and terpenoid compounds) and natural extracts like curcumin, exert broad-spectrum antibacterial activity through multi-target mechanisms—including disruption of microbial cell membranes, interference with enzymatic function, and damage to genetic material [193,194]. However, each type of these natural antimicrobial agents has its own limitations, as essential oils exhibit high volatility and poor chemical stability, resulting in limited efficacy when used directly; conversely, curcumin and other extracts suffer from low water solubility and susceptibility to photothermal degradation, making it difficult to maintain long-lasting antibacterial activity. Their incorporation into biopolymer substrates (e.g., CS, starch, PLA) enables the design of long-lasting and sustained-release antimicrobial systems. For example, gelatin–CMC–guar gum films containing green onion extract effectively suppress microbial growth [195]. Nevertheless, practical application faces challenges such as the high volatility of essential oils and limited compatibility with polymer matrices. Functional nanomaterials—including CQDs, LAP nanosheets, MOFs, and metal nanoparticles—enhance interactions with microorganisms through high specific surface area and interfacial effects [187,196]. Simultaneously, they serve as carriers to improve the dispersion, stability, and efficacy of other active ingredients. For instance, surface-modified LAP nanosheets in collagen matrices enable stable loading of active compounds, while the migration behavior and biosafety of nanomaterials through standardized assessments remain critical for practical use [142]. Selected examples of the response behavior of bio-based smart packaging materials in food quality monitoring are presented in Table 3.

### 3.2. Antioxidant and Freshness-Preserving Functions

While excellent barrier properties in bio-based packaging reduce the impact of molecular oxygen, it is the antioxidant activity, powered by the free radical-scavenging mechanisms of natural compounds like phenolics and curcumin, that plays the crucial role in delaying oxidative degradation and ensuring food preservation [187,197]. Specifically, phenolic substances neutralize free radicals by providing hydrogen atoms, while Cur captures reactive oxygen species via its conjugated double bonds [192]. For instance, incorporating tannic acid into gelatin matrices can increase DPPH radical-scavenging rates to 56% [198]. Furthermore, natural antioxidants can be effectively incorporated into packaging matrices via advanced dispersion strategies. As an example, the uniform distribution of Cur within a BC film using a Pickering emulsion system not only delivers high antibacterial efficacy (approaching 100%) but also confers radical-scavenging capacities of 27% (DPPH) and 74% (ABTS) [199]. Although synthetic nanoparticles (e.g., Se, TiO_2_, SiO_2_) exhibit alternative antioxidant mechanisms and enhanced thermal stability and mechanical properties, natural antioxidants are generally preferred for their superior biocompatibility and broader consumer acceptance [187,192,200,201].

Antioxidants are sensitive to pH, temperature, light, and time. Synergistic effects between bio-based matrices (e.g., starch, sodium alginate, PLA) and active agents can significantly enhance antioxidant and preservation efficiency. Matrices not only provide stable loading platforms for antioxidants but also prevent degradation and loss during processing or storage, through interactions like hydrogen bonds and ionic bonds. Simultaneously, the barrier properties of these matrices reduce exposure of food to oxidative triggers such as oxygen and moisture. For example, incorporating CNC into gelatin/tannic acid films allows the highly reactive hydroxyl groups on CNC surfaces to form hydrogen bonds with tannic acid. This weakens the crosslinking between tannic acid and gelatin, promoting antioxidant release, while simultaneously enhancing oxygen barrier properties by forming a dense network structure, collectively boosting preservation efficacy [198].

### 3.3. Barrier Properties

The barrier performance of bio-based smart packaging is fundamental to preserving food quality, achieved by controlling the permeation of oxygen, water vapor, light, CO_2_, volatiles, microorganisms, and lipids. This effectively mitigates oxidation, moisture uptake, photodegradation, spoilage, and flavor loss, addressing the diverse storage requirements of perishable foods.

For oxygen-sensitive products (e.g., fresh and lipid-rich foods), oxygen barriers retard lipid oxidation and microbial growth, while CO_2_ barriers help maintain internal atmosphere equilibrium to delay ripening [187]. The gas barrier properties of bio-based materials rely on molecular chain density and crystallinity, which can be enhanced through blending, nanocomposites (e.g., ordered cellulose nanocrystal layers), or multilayer designs [202,203]. For instance, CNFs form entangled networks that prolong gas diffusion paths, while starch or chitosan-based matrices—when combined with nanofillers (e.g., LAP nanosheets) or crosslinking strategies—create dense interfacial networks that block volatile transfer and flavor degradation [142,187,204,205]. Bio-inspired multilayer films further optimize performance: a hydrophobic outer layer limits oxygen ingress, while an active inner layer absorbs residual oxygen [206]. Ethylene-scavenging functionalities can also be incorporated to delay fruit and vegetable ripening and decay. For example, alkaline treated halloysite nanotubes (a-Hal) can efficiently adsorb ethylene due to their well-defined mesoporous structure [207].

Moisture barrier properties maintain humidity equilibrium within packaging by regulating vapor transmission, preventing food from softening due to moisture absorption or shriveling from dehydration. For foods requiring low humidity environments, such as grains and nuts, hydrophobic materials like acetylated cellulose reduce vapor ingress. For foods needing moderate humidity, such as fruits, vegetables, and fresh meat, hygroscopic systems are constructed using hydrophilic groups in polysaccharide substrates or porous nanomaterials. Multilayer architectures combine hydrophobic exterior layers with hydrophilic inner phases for dynamic humidity control [208]. In addition, hydrophobic antimicrobials such as essential oils, when incorporated into bio-based matrices, impose a tortuous pathway for water molecules, thereby further improving moisture barrier efficiency without compromising biodegradability.

Light exposure accelerates photooxidation of lipids, proteins, and pigments, leading to nutrient degradation, off-flavors, and discoloration [209]. The photo-oxidation process can be blocked through natural UV absorbers (e.g., bamboo leaf extracts and Cur) or nanocomposite designs (e.g., BC nanocrystals forming tortuous channels that scatter light) [210,211]. Nanoparticles like ZnO are particularly effective, as they possess a wide bandgap that enables strong UV absorption, lowering the availability of high-energy photons that initiate photooxidation. Similar UV-blocking effects can be achieved with other nanofillers, including titanium dioxide or cerium oxide. Beyond light management, bamboo extract also provides radical-scavenging activity, while metal complexes contribute photothermal antibacterial effects. Composites of high-density bio-based matrices with nanofillers further enhance light-blocking efficiency. Bio-based materials inhibit microbial invasion through physical obstruction (e.g., BC networks) and functional coatings (e.g., PHA-based layers), simultaneously providing lipid barriers to prevent oil migration and rancidity in fatty foods [212,213].

### 3.4. Controlled Release and Stimuli-Responsive Systems

Controlled-release and stimuli-responsive systems represent a research frontier in bio-based smart packaging, achieving sustained and intelligent functionality by encapsulating active substances within microcapsules or nano-carriers for targeted release under specific environmental stimuli [214,215,216,217]. Microencapsulation and nano-carrier delivery technologies serve as the foundation for the controlled release of active ingredients in bio-based packaging, effectively preventing their deactivation or concentration imbalance caused by direct food contact [218,219,220]. Bio-based carrier materials (e.g., SA, CS, starch) can form microcapsules via ion crosslinking or emulsion polymerization. For instance, embedding Artemisia annua essential oil (AAEO) within a gelatin-Arabic gum (GAG) protective shell creates shell–core microcapsules with sustained-release properties to reduce volatility, enabling prolonged antimicrobial effects [221]. Nano-carriers like nano-cellulose, zein nanoparticles, and LAP nanosheets leverage high specific surface area, excellent biocompatibility, and modifiability to achieve uniform dispersion and sustained release of active ingredients, enhancing functional persistence [142,222].

pH-, temperature-, and humidity-responsive release mechanisms leverage the sensitivity of bio-based materials to environmental parameter changes, enabling intelligent release of active ingredients that aligns with food quality degradation processes [223,224,225]. pH-responsive systems employ polyelectrolytes such as CS or HPMC, which undergo reversible structural changes in response to pH shifts induced by spoilage metabolites (e.g., organic acids or basic nitrogen compounds), triggering the controlled release of antimicrobials, indicator substances, or color changes. For instance, HPMC/SA-Cur composite films release curcumin under acidic conditions generated by spoiling pork and shrimp, providing simultaneous preservation and visual freshness signaling [184]. Temperature-sensitive systems leverage the phase-change behavior of bio-based polyester like PHA and PLA to modulate diffusion pathways of active compounds in response to temperature variations, making them suitable for cold-chain applications. Humidity response achieves switching between moisture absorption and moisture barrier functions, or humidity-triggered release of active ingredients, through the reversible transformation of hydrophilic/hydrophobic structures in bio-based materials. Under high-humidity environments, water infiltration induces matrix swelling, increasing porosity and accelerating the release of active compounds such as borneol to prevent premature or excessive emission [226]. Additionally, the combination of a hydrophobic outer layer and a hydrophilic inner layer enables unidirectional moisture transfer from the interior to the exterior based on environmental humidity gradients, reducing condensation buildup and improving preservation of moisture-sensitive produce such as cucumbers [227].

**Figure 4:**
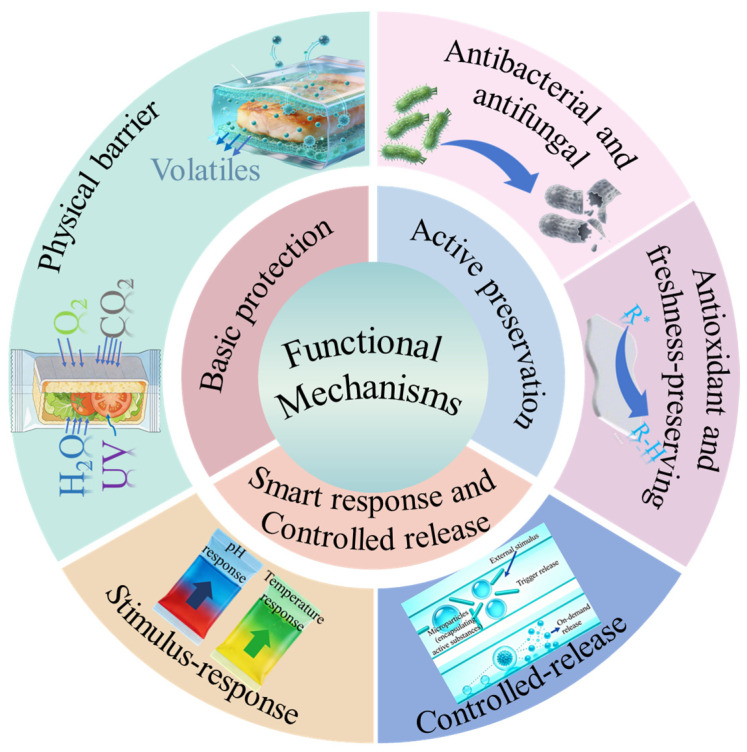
Functional mechanisms of bio-based smart packaging: baseline barrier (physical blocking, volatile control), active preservation (antioxidant, antimicrobial), controlled release, and smart responses (pH-responsive, temperature-responsive). (The decorative elements and icons in Figure 4 depicting physical barrier and controlled release were generated using AI tool (Doubao)).

In Figure 4 are summarized the functional mechanisms of bio-based smart packaging, namely baseline barrier with physical blocking and volatile control, active preservation via antioxidant and antimicrobial effects, controlled release, and smart responses including pH- and temperature-responsiveness.

**Table 3:** Examples of responsive behavior in bio-based smart packaging materials for food quality monitoring.

Basic Bio-Based Materials	Smart Components	Food Model	Behavior/Trigger	Refs.
Konjac glucomannan/CMC	ACNs	Fish	Red to yellow–green (pH: 2–12)	[228]
CNC/nanofiber cellulose	ACNs	Shrimp	Light purple–light green (ammonia and pH (1–13))	[229]
Soy protein isolate/CNC	Cur	Shrimp	Yellow–orange (ammonia and pH (3–11))	[141]
CS/polyvinyl alcohol	Shikonin and nano-ZnO	Shrimp	Dark red–dark bluish (ammonia and temperature)	[230]
Cellulose acetate	Cur	Aluminum can (water)	Yellow–red (temperature)	[132]
HPMC/Zn^2+^-SA	Cur	Pork and shrimp	Yellow–red (nitrogen and pH (2–12))	[184]
Starch	Beetroot extract	Chicken and fish	Red–pale yellow (pH: 2–12)	[99]
Laponite/caffeic acid	Cur	Shrimp	Yellow–reddish brown (ammonia and pH (3–11))	[142]
PLA/nanofiber cellulose	ACNs	Cherry tomato	Purple–greenish yellow (pH: 2–14)	[231]

## 4. Intelligent Sensing, System Integration, and Sustainability Assessment

### 4.1. Intelligent Sensing and Responsiveness

Intelligent sensing and response constitute the core of bio-based smart packaging, enabling real-time food quality monitoring through three pivotal technologies. Freshness colorimetric indicators (e.g., pH-responsive natural pigments, gas-responsive dyes) enable rapid visual feedback on spoilage, providing qualitative information [232,233,234,235,236,237,238,239]. Bio-based electrochemical and conductive sensors utilize conductive composite materials based on biopolymers like CS and sericin to build flexible printable sensing platforms for quantitative detection of key quality parameters [240,241]. Functional nanomaterial-enabled sensing technologies enhance detection selectivity through the designable channels of MOFs and COFs, while boosting detection sensitivity via nano-enzyme-catalyzed signal amplification [242,243,244,245,246]. Aligned with green sustainability principles, these technologies collectively advance smart packaging from qualitative indication toward precise, sensitive quantitative monitoring.

### 4.2. Integration of Smart Packaging into Next-Generation Food Systems

Smart packaging is evolving from a passive barrier into an active, decision-making core for next-generation food systems. Functioning first as a data interface, it integrates bio-based sensors with Near Field Communication (NFC)/Radio Frequency Identification (RFID) technology to build real-time monitoring systems capable of interacting with smart devices [247,248,249,250,251,252,253,254,255], progressing to IoT- and blockchain-enabled traceability [256,257,258,259,260,261,262], and culminating in autonomous system intelligence for dynamic quality control and risk alerts, thereby supporting a more efficient, transparent, and resilient food system [263,264,265,266].

### 4.3. Safety Standards and Lifecycle Considerations

The innovation and scaling of bio-based smart packaging must advance within a framework of safety and sustainability, achieving a unified balance of functionality, safety, and environmental value. The primary consideration is food contact safety [267,268,269,270]. A rigorous evaluation of migration behavior and toxicological risks of smart components (e.g., nanoparticles and pigments) is required, along with establishing corresponding testing protocols. Systematic research must be conducted on their migration behavior, degradation products, and potential toxicological risks in various food simulants to ensure long-term consumption safety. Secondly, biodegradability and end-of-life scenarios must be clearly defined. Not all bio-based materials degrade rapidly in natural environments. The compostability conditions (industrial or household composting) must be specified. Materials should degrade completely under specific conditions (e.g., industrial composting), with degradation rates aligned to shelf life to prevent secondary pollution from microplastics or ecotoxicity. Finally, life cycle assessments (LCAs) must quantify environmental performance [271,272]. By conducting multidimensional comparisons with traditional plastic packaging, the advantages and limitations of bio-based smart packaging in energy consumption, pollutant emissions, and resource utilization can be clarified. This provides data support for material optimization and process improvements, driving continuous enhancement of environmental sustainability.

## 5. Application Scenarios for Bio-Based Smart Packaging Materials

### 5.1. Fresh Food and Produce

Fresh produce (such as fruits, vegetables, meat, and seafood) is highly perishable with a short shelf life, demanding exceptional packaging performance in freshness preservation and quality monitoring [273,274,275,276]. This makes it a core application scenario for smart packaging. Bio-based smart packaging integrates long-lasting freshness preservation with freshness sensing capabilities, enabling targeted solutions to address these. For post-harvest produce, respiration and transpiration can disrupt gas equilibria (e.g., O_2_, CO_2_), while ripening-related gases like ethylene accelerate overripening and decay [277,278,279]. Packaging must therefore regulate gas and water vapor transmission while incorporating adsorbents—such as functionalized MOFs—to selectively trap spoilage-related gases and delay deterioration [280,281]. Furthermore, these integrated materials can serve as smart carriers for controlled release of antimicrobial agents and provide visual freshness feedback. For meat and seafood, where spoilage raises pH and releases amines, packaging can incorporate pH-responsive films—for example, those containing ACNs—which offer visual freshness feedback through distinct color changes as pH shifts from acidic to alkaline [282,283].

### 5.2. Convenience Food

The market for convenience foods—such as instant noodles, self-heating meals, and snacks—is large, demanding packaging that ensures safety and quality while improving user convenience and experience. Bio-based smart packaging materials show unique value in this sector. Edible packages (EPs), including films or coatings made from biopolymers such as proteins, polysaccharides, or lipids, can serve directly as seasoning packet liners, realizing the concept of “packaging as food” while reducing plastic use [284,285,286]. Water-soluble bio-based films can be used in heating packs for self-heating meals, dissolving upon contact with water to release heating agents while combining safety with convenience. For microwave-ready products, packaging must be heat resistant and capable of integrating heat-sensitive labels to verify thorough heating. Furthermore, by integrating sensors and QR codes via printed electronics, these materials can provide traceability and nutritional guidance, boosting transparency and engagement.

### 5.3. Functional Food

To meet the stringent requirements of functional foods for stable protection of active ingredients, maintenance of efficacy, and effective delivery, bio-based smart packaging offers systematic solutions. High barrier bio-based packaging (such as polyester) effectively blocks oxygen and light, preventing oxidation and degradation of light-sensitive nutrients [84,287]. For live microbial products, integrated oxygen scavenging and desiccants create anaerobic, low-humidity microenvironments to sustain activity. For nutrient fortification and delivery, edible films loaded with nutrients (e.g., probiotics, fiber) enable direct nutritional supplementation [288,289]. Combined with stimuli-responsive release systems (e.g., tear-activated), protective agents or flavor compounds can be precisely released during consumption, enabling on-demand delivery. Additionally, this packaging further employs smart controlled-release and selective barrier mechanisms to continuously protect active ingredients during storage and transport, preventing oxidation and moisture-induced degradation.

### 5.4. Paper-Based Food Packaging

**Figure 5:**
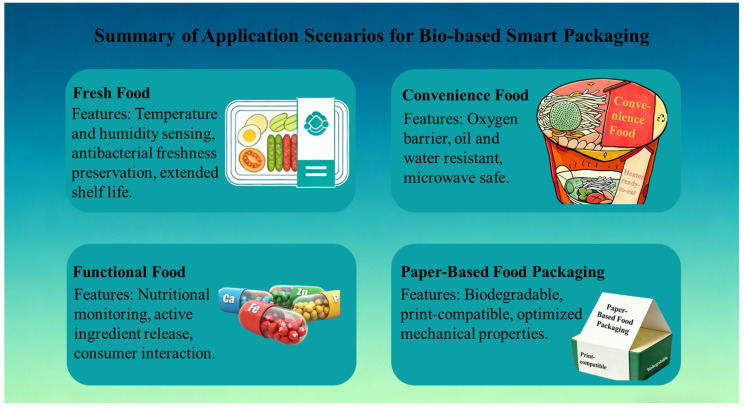
Application scenarios of bio-based smart packaging across major food categories: fresh produce (temperature/humidity sensing, antibacterial, shelf life extension); convenience foods (oxygen barrier, oil/water resistance, microwave compatibility); functional foods (nutrition monitoring, active ingredient release, consumer interaction); and paper-based packaging (biodegradability, printability, mechanical optimization). (The decorative elements and icons in Figure 5 depicting functional food were generated using AI tool (Doubao)).

Owing to its recyclability, biodegradability, and absence of microplastic risks, paper-based packaging is a key sustainable alternative for next-generation food systems [290,291,292,293]. Its intelligent properties are primarily achieved through surface functionalization via coating or impregnation, as well as structural integration technologies [294,295]. For instance, coating paper surfaces with bio-based barriers like chitosan or starch derivatives significantly enhances water and oil resistance. Further embedding smart indicators (e.g., Cur, ACNs) or functional nanomaterials (e.g., ZnO, MOFs) into these coatings endows packaging with freshness monitoring or antimicrobial capabilities. As exemplified by ZIF-67/shellac composite coatings, such integration can maintain high barrier performance while enabling rapid gas sensing (e.g., ammonia), significantly expanding the functional scope and application value of paper-based smart packaging [296,297]. Figure 5 illustrates the functional design of bio-based smart packaging across four key application areas, showcasing the targeted alignment between material capabilities and specific usage requirements.

## 6. Challenges and Future Perspectives

### 6.1. Major Challenges

Bio-based smart packaging presents a promising strategy to address current challenges in food safety, promote sustainable development, and reduce food waste. Current research focuses on developing smart packaging materials capable of responding to multiple indicators, such as humidity, pH, and temperature, enabling real-time visual monitoring of food quality. However, scaling this technology for widespread application still faces multiple challenges.

1. The long-term stability and reliability of smart responses require improvement. Natural indicators (such as ACNs) are prone to degradation by light, heat, and oxygen exposure, compromising the reversibility and precision of color changes in complex food systems and during extended storage, while sensor signals drift over time. Similar stability issues also affect other natural active agents—essential oils are volatile and prone to oxidation, while antimicrobial peptides can be degraded by proteolytic enzymes—highlighting a common challenge for incorporating diverse natural compounds into smart packaging. Additionally, the controlled release and matrix compatibility of functional nanomaterials (e.g., nanoparticles, MOFs) also require further optimization.
2. Achieving synergistic optimization among performance, cost, and sustainability proves challenging, with these factors mutually constraining commercialization. High-performance bio-based materials (e.g., PHA, nano-cellulose) and smart components (e.g., MOFs) often carry high costs, while their production may conflict with green objectives due to energy and chemical inputs.
3. Bottlenecks exist in translating laboratory prototypes to industrial-scale applications. Most smart packaging preparation processes (e.g., precision coating, electrospinning) are complex and inefficient, lacking mature mass production technologies and supporting processes. Concurrently, the absence of unified performance standards and safety assessment protocols severely hinders commercialization.
4. Finally, regulatory compliance poses a major obstacle. Approval processes for novel bio-based packaging are time-consuming and costly, particularly due to limited toxicological data and health risk assessments. Furthermore, the incorporation of smart functional components raises concerns about direct food contact safety. While most studies indicate low cytotoxicity, concerns over genotoxicity and nanoparticle migration persist, affecting consumer and regulatory acceptance.

Addressing these interrelated challenges is essential to fully realize the potential of bio-based smart packaging in next-generation food systems.

### 6.2. Future Outlook

The future development of bio-based smart packaging will focus on material functional upgrades, evolving toward biomimetic, adaptive, and system-integrated directions to drive packaging from single-function to intelligent, multi-functional integration. Near-term development focuses on optimizing materials and processes: enhancing active component stability through advanced encapsulation and curing; tuning the barrier, mechanical, and thermal properties of substrates via customized chemical or enzymatic modification; and utilizing low-cost, renewable feedstocks like agricultural waste to develop green processing routes. Furthermore, integrating with established technologies can reduce costs and increase consumer acceptance, balancing performance with economic and environmental goals. Concurrently, functional stability must be strengthened, and safety standards must be established to ensure public trust. This includes employing encapsulation or covalent fixation to prevent indicator migration and enhance durability, alongside developing material-specific toxicity protocols and regulatory frameworks to define safety limits and build public trust. Additionally, rigorous evaluation with large, diverse sample sets is needed to minimize false positives and optimize the packaging’s effectiveness in reducing food waste.

The medium-to-long-term evolution of bio-based smart packaging will progress toward higher-level intelligence by developing self-healing, adaptive biomimetic materials, enabling scalable, low-cost production through process integration, and transforming packages into IoT nodes. These intelligent systems fuse real-time food data with supply chain AI, thereby enabling a critical shift from passive monitoring to predictive regulation and autonomous control.

## 7. Conclusions

Bio-based smart packaging combines renewable feedstocks with responsive functionalities, shifting packaging from passive containment to active quality monitoring and supply chain information nodes. The market reflects this shift: the smart food packaging sector is projected to reach USD 48.97 billion by 2028 (CAGR 4.49%). At the material level, natural polymers, biopolymers, and bio-based polyesters provide the structure–property foundation. Among these, cellulose and its derivatives have drawn the strongest research interest over the past five years, confirming their leading role as renewable, versatile materials. Beyond passive barriers, these materials enable active intervention through core functions—active preservation (antimicrobial, antioxidant, controlled release) and intelligent sensing (pH-responsive indicators, IoT integration). For instance, chitosan with essential oils offers proactive preservation, while pH-sensitive anthocyanins visually indicate spoilage. Smart release systems triggered by pH, temperature, or gases shift from continuous to on-demand action. On the informational front, packaging acts as an in situ sensing terminal. Sensing technologies have evolved from colorimetric indicators to electrochemical sensors enhanced by nanomaterials (e.g., MOFs, nanozymes). Integration with IoT and blockchain bridges physical and digital supply chains, enabling full traceability.

Challenges remain with long-term stability, cost–sustainability trade-offs, scalability, and regulation. Future work should prioritize biomimetic design, system integration, and AI-driven analytics toward resilient, self-adaptive food systems. Ultimately, this evolution supports safer, more transparent, efficient, and sustainable food systems.

## Figures and Tables

**Table 2:** Improvements and performance of bio-based polyesters smart packaging.

Strategy	Modification	Advantages	Refs.
Nanocomposite materials	Coaxial electrospinning encapsulates cinnamaldehyde (CMA) and tea polyphenols (TPs) within PLA nanofibers.	Significantly enhances mechanical and barrier properties; sequential release of TP and CMA achieves optimal synergistic antimicrobial efficacy.	[179]
Polymer blending; functional additives	Cur acts as a responsive indicator and antioxidant within a PLA/polypropylene carbonate (PPC) matrix.	Monitors the freshness of shrimp and other foods; PPC enhances water and oxygen barrier, while Cur provides antioxidant activity.	[180]
Functional additives	Caffeic acid and green tea extract are incorporated into PLA as antioxidants.	Enhanced thermal oxidation stability; provides intuitive, intelligent indicators of degradation during oxidation.	[181]
Functional additives	Formic acid enables the uniform dispersion of hydrophilic tannic acid within poly(hydroxybutyrate-co-valerate) (PHBV) matrix.	Tannic acid delivers antioxidant, UV shielding, and high barrier performance; intelligently signals spoilage; extended shelf life.	[182]
Plasticizers; functional additives	PLA-PHB matrix is enhanced with glycerol monolaurate (GML) for plasticization and cinnamaldehyde for antimicrobial activity.	Excellent mechanical properties and strong antimicrobial activity; preserves freshness.	[183]
Layer-by-layer assembly	Introducing carboxylated cellulose nanofibers as nano-reinforcing phases into PEF.	Mechanical and barrier properties significantly outperform pure PEF film; extended shelf life.	[169]
Nanocomposite materials	Utilizing PEF as a matrix, nano-TiO_2_ as a functional additive, and PMDA as a chain extender.	Enhances overall performance; UV/blue light shielding and antibacterial; inhibits Ti migration.	[175]

## Data Availability

No new data were created or analyzed in this study. Data sharing is not applicable to this article.

## Abbreviations

CNF	Cellulose nanofiber	CNC	Cellulose nanocrystal
MC	Methyl cellulose	HPMC	Hydroxypropyl methylcellulose
CA	Cellulose acetate	BC	Bacterial cellulose
EC	Ethyl cellulose	CMC	Carboxymethyl cellulose
CS	Chitosan	CMCS	Carboxymethyl chitosan
PVA	Polyvinyl alcohol	PEF	Poly (ethylene 2,5-furandicarboxylate)
ACN	Anthocyanin	PET	Polyethylene terephthalate
SA	Sodium alginate	MOF	Metal–organic framework
Cur	Curcumin	COF	Covalent organic framework
PHA	Polyhydroxyalkanoate	PLA	Polylactic acid
PHB	Polyhydroxybutyrate	PHV	Polyhydroxy-valerate
LAP	Laponite	EP	Edible package
